# Novel resorbable bone wax containing β-TCP and starch microspheres for accelerating bone hemostasis and promoting regeneration

**DOI:** 10.3389/fbioe.2023.1105306

**Published:** 2023-01-19

**Authors:** Qiangqiang Duan, Huiling Liu, Lixia Zheng, Daozhang Cai, Guangxin Huang, Yu Liu, Rui Guo

**Affiliations:** ^1^ Guangzhou Beogene Biotech Co., Ltd., Guangzhou, China; ^2^ Department of Joint Surgery and Sports Medicine, The Third Affiliated Hospital of Southern Medical University, Guangzhou, China; ^3^ The Third School of Clinical Medicine, Southern Medical University, Guangzhou, China

**Keywords:** resorbable bone wax, bone regeneration and hemostatic, alkylene oxide copolymers, *β*-tcp, starch microsphere

## Abstract

Traditional non-resorbable bone wax has been used in clinical surgery for more than 100 years. However, residual bone wax has been proven to cause numerous complications. In this study, a novel resorbable bone wax was designed to overcome the disadvantages of traditional non-resorbable bone wax. Alkylene oxide copolymers were used as the main component of resorbable bone wax; additionally, *β*-tricalcium phosphate and starch microspheres were added to enhance bone regeneration and hemostatic ability. This novel resorbable bone wax has a high potential for clinical translation and is expected to be developed as a substitute for traditional bone wax.

## 1 Introduction

Bone is a highly dynamic vascularized tissue. Osseous hemorrhage occurs during surgery or when a badly fractured bone is difficult to control. Therefore, bone wax was introduced as a mechanical bone hemostatic agent by V. Horsley in 1886. Bone wax is a waxy hemostatic agent that is used to control local bone bleeding during surgery (Schonauer et al., 2004; Achneck et al., 2010; Das, 2018). It coats the cutting area of the bone to form an impenetrable mechanical barrier, blocking blood flow from damaged vessels in bone and allowing clotting to occur. Owing to its low cost and good operability, the formulation of bone wax has not changed much since 1924 (Schonauer et al., 2004). The materials for preparing bone wax are still beeswax, isopropyl palmitate, and softening agents (Schonauer et al., 2004; Achneck et al., 2010). However, clinical cases have reported numerous complications related to its non-resorbable material, such as failed bone healing (Ozerdem et al., 2013), foreign body reaction (Eser et al., 2007; Thangamathesvaran et al., 2019), granuloma growth (Lavigne et al., 2008), thrombosis (Chun et al., 1988), infection (Choi and Yang, 2017), nerve damage (Katre et al., 2010), migration (Fahradyan et al., 2018; Zhou et al., 2019a), and extrusion (Baird et al., 2018).

To avoid the problems associated with traditional bone wax, new resorbable synthetic substitutes have been developed, including PEG/microfibrillar collagen composites (Orgill et al., 2015), alkylene oxide copolymers (Salim, 2018; Kim et al., 2020; Choi et al., 2021), hydroxyapatite/polylactic acid (Tham et al., 2018), alkylene oxide copolymers/carboxymethylcellulose sodium salt composites, poly (ethylene glycol)-poly (propylene glycol) copolymer/starch composites (Suwanprateeb et al., 2014), modified calcium sulfate cement (Zhou et al., 2018), and chitosan (Chen et al., 2015). However, absorbable materials might cause allergic reactions and retardation of bone regeneration (Schonauer et al., 2004). These substitutes not only offer new bone hemostatic agents to avoid the negative biological effects of bone wax, but also attempt to promote bone regeneration.

In this study, we prepared novel resorbable bone waxes based on alkylene oxide copolymers, *β*-tricalcium phosphate (β-TCP), and starch microspheres. Alkylene oxide copolymers are water-soluble copolymers that are biocompatible, chemically inert, non-metabolizable, and resorbable (Michael et al., 2002). Therefore, alkylene oxide copolymers have been widely use in medical and pharmaceutical fields (Wang et al., 2001). Certain copolymers in the family have similar physical properties to bone wax but the absorbable property avoids the negative biological effects. *β*-TCP is a biodegradable ceramic material that has been commonly used in orthopedics as implant material (Ogose et al., 2005). It is known to be biocompatible, bioactive, and osteoconductive and has shown a favorable substitution rate in standardized bone defects (Buser et al., 2010). Starch microspheres are biocompatible materials with large surface areas that increases their water absorption capacity (Chao et al., 2017; Yu et al., 2017). By absorbing fluid from blood, starch microspheres increase the concentration of platelets and clotting proteins (Björses et al., 2011). Consequently, starch microspheres are considered to be a biodegradable (Malafaya et al., 2007), biocompatible, and cost-effective (Suwanprateeb et al., 2013) supplementation to hemostatic agents.

In this present work, a novel resorbable bone wax, based on the combination of alkylene oxide copolymers, *β*-TCP, and starch microspheres, was prepared to simultaneously achieve rapid hemostasis and enhanced bone regeneration. This study evaluates its compressive strength, *in vitro* degradation performance, and cytotoxicity properties. The *in vivo* performance of the resorbable synthetic bone waxes and traditional bone wax was compared, including hemostasis, histopathology, and bone regeneration, in a rabbit tibia defect model. Compared with the commercial non-absorbable Braun Bone Wax, this new absorbable bone wax showed a comparable hemostatic effect and better bone healing effect; therefore, it is expected to be transformed into a product to replace traditional bone wax.

## 2 Materials and methods

### 2.1 Materials

Calcium nitrate tetrahydrate (Ca(NO_3_)_2_·4H_2_O), ammonium phosphate dibasic ((NH_4_)_2_HPO_4_), and Span 80 were purchased from Sinopharm Chemical Reagent Co., Ltd. (Shanghai, China). Ammonium hydroxide solution (NH_3_·H_2_O), anhydrous ethanol, soluble starch, trisodium trimetaphosphate, poly (ethylene glycol) (average Mn = 4,000), and olive oil were purchased from Macklin Biochemical Co., Ltd. (Shanghai, China).

### 2.2 Synthesis of *β*-tricalcium phosphate

β-TCP used for preparing bone wax was synthesized through chemical precipitation and the hydrothermal method (Grigoraviciute-Puroniene et al., 2017). Ca(NO_3_)_2_·4 H_2_O aqueous solution (0.3 M) was added to (NH_4_)_2_HPO_4_ aqueous solution (0.2 M) dropwise with continuous moderate stirring at 40°C for 24 h and the pH value was maintained at 7 using ammonia solution. The obtained precipitate was vacuum filtrated, washed with anhydrous ethanol, and then dried in an oven at 120°C for 12 h. The *β*-TCP powder was finally obtained by calcination in a muffle furnace at 800°C for 2 h.

### 2.3 Preparation of starch microspheres

Starch microspheres for resorbable bone wax preparation were synthesized using the reversed phase suspension method. A total of 10 g of soluble starch was pasted by heating 10% slurry in distilled water at 95°C with thorough stirring and the pH was maintained at 10. Next, 0.8 g of trisodium trimetaphosphate and 0.8 g of poly (ethylene glycol) dissolved in 30 mL of distilled water were added to the pasted starch and homogenized. Then, 2 g of Span 80 was added in 150 mL of olive oil and kept at 50°C with continuous stirring; the starch mixture was then added dropwise with stirring for 6 h to allow crosslinking. The crosslinked starch microspheres were obtained by centrifugation and then washed with anhydrous ethanol and distilled water alternately three times. The product was finally obtained after lyophilization.

### 2.4 Preparation of resorbable bone wax

Resorbable bone waxes of various compositions were prepared according to the formulations presented in Table 1. Proprietary formulations of alkylene oxide copolymers, *β*-TCP, and starch microspheres were melted by mixing at 80°C for 1 h at different ratios, designated as a, b, c, and d. All samples were sterilized by gamma irradiation at 25–40 kGy before assessment.

**TABLE 1:** Different compositions of resorbable bone wax.

Sample	Alkylene oxide copolymers	Starch microspheres	β-TCP
a	5	0	0
b	5	0.2	0
c	5	0	0.2
d	5	0.2	0.2

### 2.5 Characterizations of *β*-TCP and starch microspheres

The phase structure of synthesized *β*-TCP was evaluated using an x-ray diffractometer (XRD, miniflex600, Rigaku Corporation, Japan) with a scanning rate of 2° 2*θ* per min over a range of 2*θ* = 10°–90°. Transmission electron microscopy (TEM, TECNAI G2 Spirit TWIN, Thermo Fisher Scientific Inc., United States) was used to determine the size and morphology of the *β*-TCP powders.

The crosslinked starch microspheres were analyzed using Fourier transform infrared (FTIR, Nicolet iS50 + iN10, Thermo Fisher Scientific Inc., United States) spectra and scanning electron microscopy (SEM, CB 340, Carl Zeiss Microscopy GmbH, Oberkochen, Germany).

### 2.6 Physical characterizations of resorbable bone wax

The compressive strength of the samples was measured using a universal testing machine (AG-1, Shimadzu Corporation, Japan) with a speed of 1 mm/min. Cuboidal samples (30 mm × 20 mm × 5 mm) were deposited in 0.01 M phosphate buffered saline (pH 7.3–7.4) at a ratio of 2.5 g/25 mL to study the immersion performance of resorbable bone wax in an aqueous environment under a constant temperature of 37°C.

### 2.7 Cytotoxicity assessment of resorbable bone wax

Rat bone marrow mesenchymal stem cells (rBMSCs) were used for cytotoxicity assessment. Sterilized samples were placed in six-well plates (the concentration of the cell suspension was 2 × 10^4^ cells/sample) and incubated at 37°C in a 5% CO_2_ incubator. The medium was changed every other day during incubation. The mass ratio of medium to sample was 10:1. At 1, 3, 5, and 7 days, cell viability was assessed using a Cell Counting Kit-8 (CCK-8, Dojindo Laboratories, Japan) assay following the manufacturer’s instructions. The optical density (OD) was measured at a wavelength of 450 nm using a microplate reader (352 Multiskan MS, Thermo Labsystems Inc., United States) and corrected by subtracting the OD from the control group (cells without samples).

### 2.8 *In Vitro* osteogenic differentiation and cell migration ability

Alkaline phosphatase (ALP) activity is considered to be an early osteogenic differentiation marker (Wang et al., 2011). The ALP activity of different resorbable bone wax was assessed using an ALP assay kit (R&D Systems, Inc., United States). The rBMSCs were seeded on six-well plates at 2 × 10^4^ per well with bone wax and incubated at 37 °C in a 5% CO_2_ incubator. At 1, 3, 5, and 7 days, the culture medium was taken from the well and rinsed three times with PBS. An aliquot of 200 μL of the lysis buffer supplied with the kit was added to each sample, which was then homogenized and sonicated for 4 min. To assess ALP activity, 50 μL p-nitrophenyl phosphate liquid substrate was added. The reaction was terminated with sodium hydroxide solution 15 min after incubation at 37°C. All solutions were collected and measured spectrophotometrically at 405 nm. The ALP activity of specimens was calculated according to the standard curve.

The effect of resorbable bone wax on cell migration was assessed by scratch wound assays. Human umbilical vein endothelial cells (HUVECs) were seeded on 48-well plates at a density of 3×10^4^ cells per well and incubated at 37°C in 5% CO_2_ for 24 h to create confluent monolayers. The cell monolayer was carefully scratched with sterile 200-μL pipette tips. Following scratch injury, cell debris was washed off with PBS and the culture medium was replaced with 200 µL of leach liquor. To measure cell mobility, the cells were photographed using an optical microscope at random fields 0, 4, and 24 h after scratching. The wound area was quantified through measurement of the photographic images using IPP 6.0 software.

### 2.9 *In Vivo* evaluation of hemostasis and bone regeneration

*In vivo* hemostasis and bone regeneration performance of sample d was evaluated in a rabbit tibia defect model. Commercial bone waxes (B. Braun Surgical, SA, Germany) were used for comparison. The animal experiment was performed at the Guangdong Laboratory Animals Monitoring Institute and approved by the internal Institutional Animal Care and Use Committee.

Twenty-four male New Zealand rabbits, weighing approximately 2.7 kg each, were randomized into three groups. Eight rabbits in each group received an implantation of commercial bone wax or resorbable bone wax. No substances were applied to the control group. Animals were anesthetized through intravenous injection of 3% pelltobarbitalum natricum solution (40 mg/kg, Merck KGaA, Germany). Right hind limbs of experimental animals were shaved and cleaned with iodine tincture. Surgery was performed along the midline to expose the tibiae and a 4.2-mm circular defect 3 cm below the knee joint was created using a medical electric drill (ZAZJ-I, Shanghai Ziai Medical Device Co., Ltd., China). Bone waxes were applied to the defects for hemostasis. The time span up until bleeding stopped and bleeding volume were recorded (up to 5 min). Wounds were then closed in multiple layers. After surgery, the animals were given ampicillin sodium (20 mg/kg, Jiangxi Jinkangjia Biochemical Pharmaceutical Co., Ltd., China) and tolfedine (0.1 mL/kg, Vetoquinol S.A., France) daily for 3 days. During the test period, animals’ appearance and physical signs were observed once a day.

Four rabbits from each group were euthanized at 6 and 12 weeks after surgery. Right hind limb tibiae were removed and observed grossly and then scanned using micro-computed tomography (micro-CT, Aloka Latheta LCT200, Hitachi, Ltd., Japan). Bone samples were fixed in 10% buffered formalin (pH 7.4) for 48 h at 37°C and decalcified in 10% EDTA for 4 weeks at room temperature. After dehydration with an ascending alcohol gradient, samples were embedded in paraffin and sectioned. Histological evaluation of treated bone samples was carried out by hematoxylin and eosin (H&E) staining, Masson staining, osteocalcin (OCN) immunostaining, safranin O staining, and Fast Green staining, followed by analysis using an optical microscope (DM3000LED, Leica Microsystems, German).

### 2.10 Statistical analysis

All tests conducted in this study used at least three parallel samples (*n* = 3). Data are mean ± standard deviation (SD).

## 3 Results

### 3.1 Characterization of *β*-TCP and starch microspheres

**FIGURE 1:**
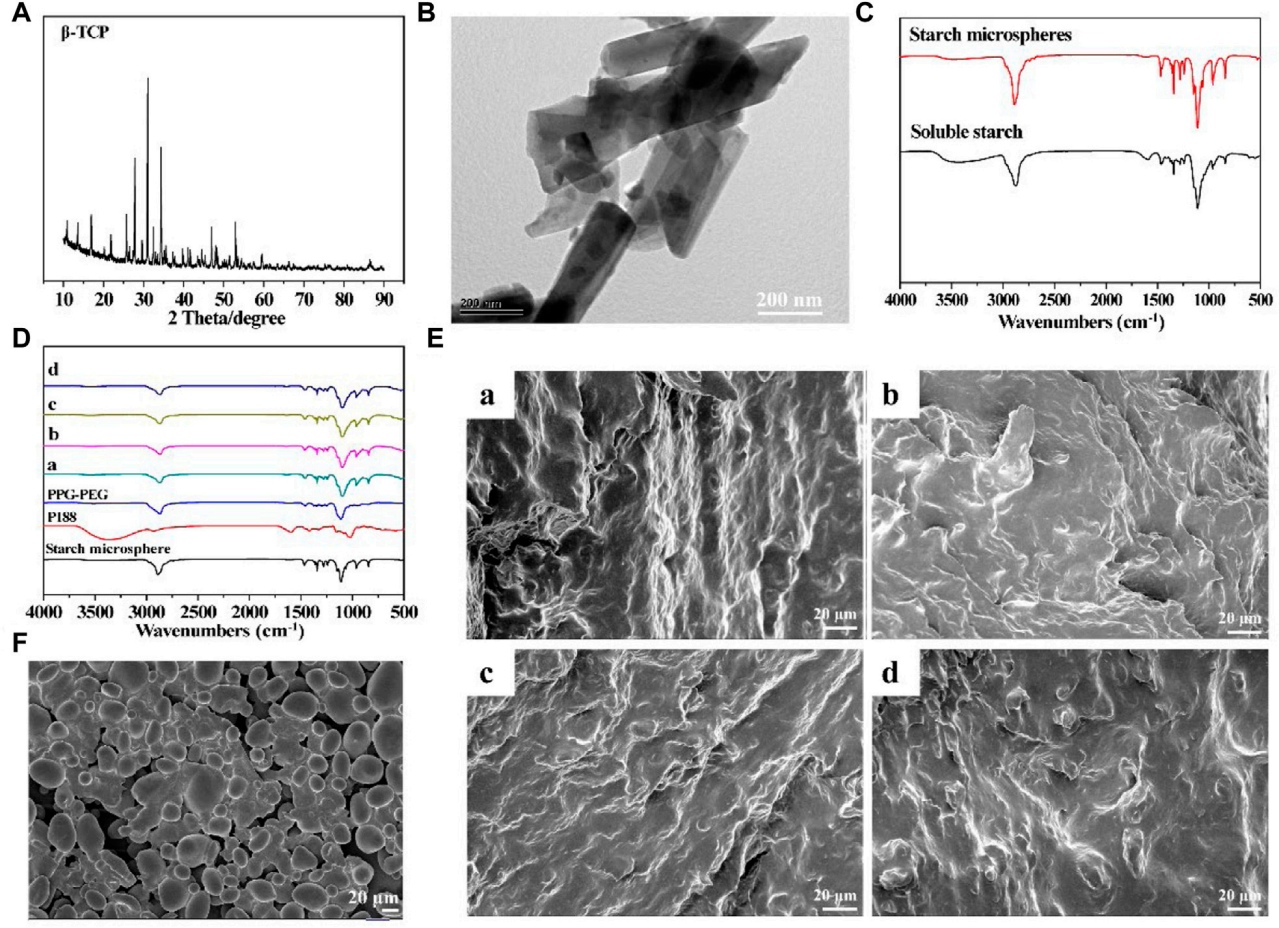
Characterization of prepared resorbable bone wax **(A)** XRD spectrum of *β*-TCP. **(B)** TEM image of *β*-TCP. **(C)** FTIR spectra of soluble starch and starch microspheres. **(D)** FTIR spectra of bone waxes with different components. **(E)** Morphology of bone waxes a, b, c and d. **(F)** SEM image of starch microspheres.

β-TCP and starch microspheres were synthesized as functional dopants to prepare the resorbable bone wax. The crystal structure of *β*-TCP was characterized using XRD and the pattern of synthesized *β*-TCP is shown in Figure 1A. Compared with the standard PDF card (JCPDS reference 01–070-2065), the successful synthesis of *β*-TCP nanoparticles could be observed (Tangboriboon et al., 2019). Additionally, the morphology of the *β*-TCP nanoparticles was examined using TEM (Figure 1B). Stick-like crystals 500 nm in length and 200 nm in width could be clearly observed.

Starch microspheres were obtained by crosslinking soluble starch, and the FTIR spectra are shown in Figure 1C. Starch microspheres and soluble starch showed comparable FTIR spectra due to their similar molecular structure. However, the wide absorption band at 3,489 cm^−1^ assigned to O-H stretching of starch microspheres was significantly weakened and shifted to a higher frequency compared with soluble starch (3,460 cm^−1^), which was thought to probably be a result of the crosslinking reaction that introduced the phosphate groups to form hydrogen bonds with the hydroxyl groups. However, the adsorption peaks of *p*=O and P-O-C did not appear in the FTIR spectrum of starch microspheres due to low crosslinking.

The alkylene oxide copolymers for preparing resorbable bone wax were poloxamer 188 (P188), polyethylene glycols (PEG), and polypropylene glycols (PPG). FTIR spectra of the resorbable bone wax prepared according to the proportions in Table 1 are shown in Figure 1D. The four different resorbable bone waxes (a, b, c, and d) all showed obvious absorption peaks of PPG-PEG and starch microspheres. The absorption peaks of P188 could not be clearly observed, probably due to the relatively lower ratio.

The morphology of starch microspheres is shown in Figure 1F. Smooth spherical microparticles with a diameter of approximately 20 μm were observed. Starch microspheres were found to be aggregated. The phenomenon of aggregation was mainly caused by the reversed phase suspension method during preparation, which was in accordance with previous reports (Li et al., 2009; Peng et al., 2011). The microstructure of resorbable bone waxes were also observed using SEM and are shown in Figure 1E. All four groups displayed a rough and tight structure, which was beneficial for sealing ruptured blood vessels at the bone injury site.

### 3.2 Physical properties of resorbable bone wax

The absorbable bone wax prepared by this ratio had strong plasticity and was easy to manipulate; adding too few hemostatic starch microspheres weakens the hemostatic effect and adding too much makes the absorbable bone wax hard and difficult to manipulate. At the same time, adding too little β-TCP weakens the bone repair effect and adding too much makes the absorbable bone wax hard and less adhesive, making it difficult to manipulate.

**FIGURE 2:**
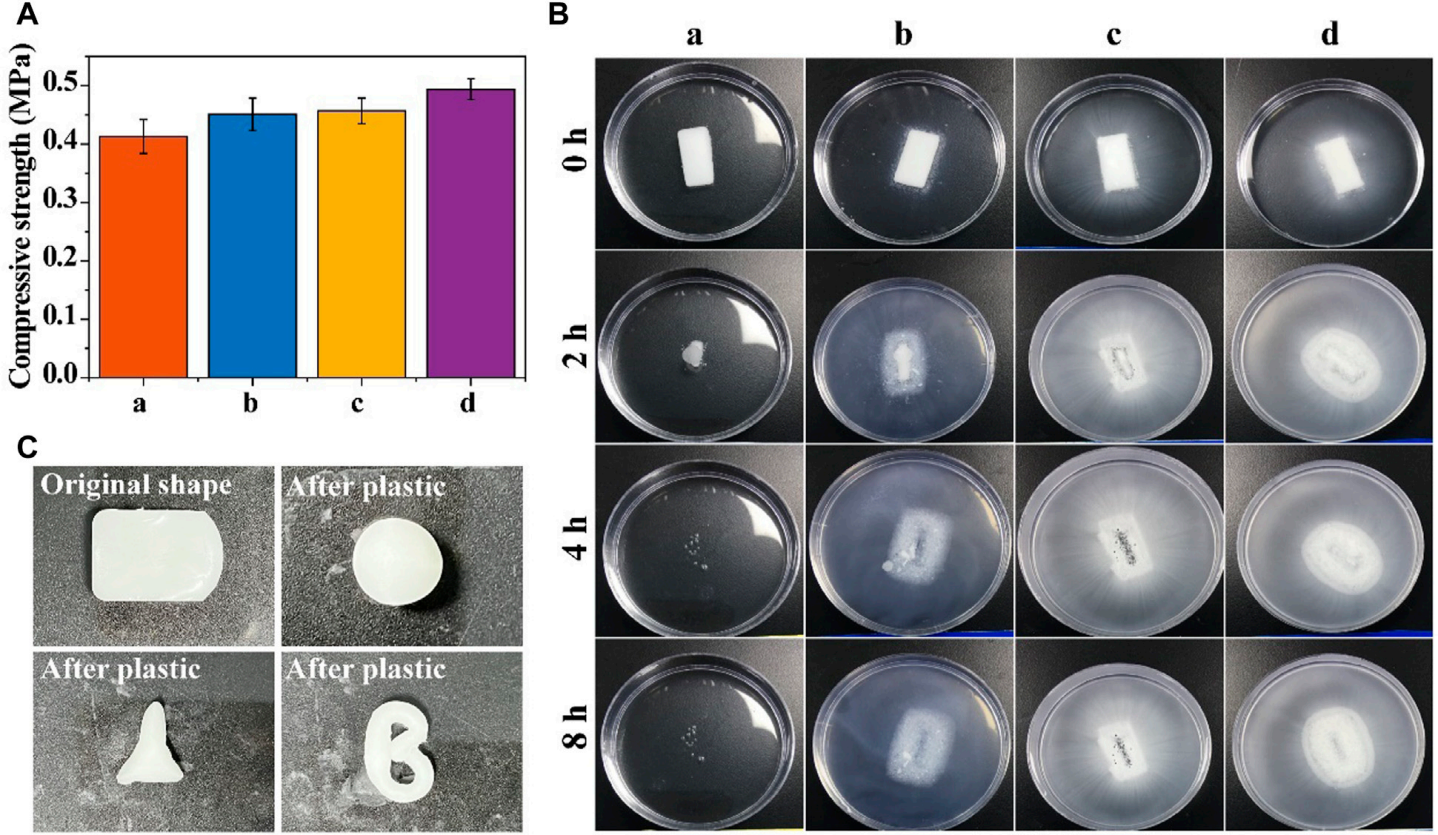
Physical properties of resorbable bone wax. **(A)** Compressive strength of bone waxes with different components. **(B)** Solubility of bone waxes in PBS buffer (0.01 M, pH 7.3–7.4). **(C)** Plasticity images of bone waxes a, b, c, and d

The compressive strengths of the different resorbable bone waxes are compared in Figure 2A. The addition of starch microspheres and *β*-TCP increased the mechanical properties of samples, with a compressive strength of 0.384–0.442 MPa for sample a, 0.423–0.479 MPa for sample b, 0.435–0.479 MPa for sample c, and 0.476–0.512 MPa for sample d. Although the addition of *β*-TCP and starch microspheres enhanced the compressive strength of the bone wax, it did not affect the usability. After being rubbed by hand, the bone wax gradually became soft and could be reshaped into different shapes according to need (Figure 2C).

Alkylene oxide copolymers can be thoroughly dissolved in water and were chosen to fabricate the resorbable bone wax. Figure 2B shows the degradation ability of the resorbable bone waxes a, b, c, and d. The experiment was conducted in an aqueous environment (PBS buffer). Resorbable bone wax only consisted of soluble alkylene oxide copolymers dissolved for approximately 4 h in PBS without any residue. The addition of starch microspheres or *β*-TCP to bone wax did not affect the disintegration of the substances. Absorbable bone waxes b, c, and d disintegrated over the same 4 h and released the starch microspheres and/or *β*-TCP particles into the surrounding solution. This indicates that bone waxes composed of alkylene oxide copolymers, *β*-TCP particles, and starch microspheres are totally resorbable.

### 3.3 *In Vitro* cytotoxicity of resorbable bone wax

**FIGURE 3:**
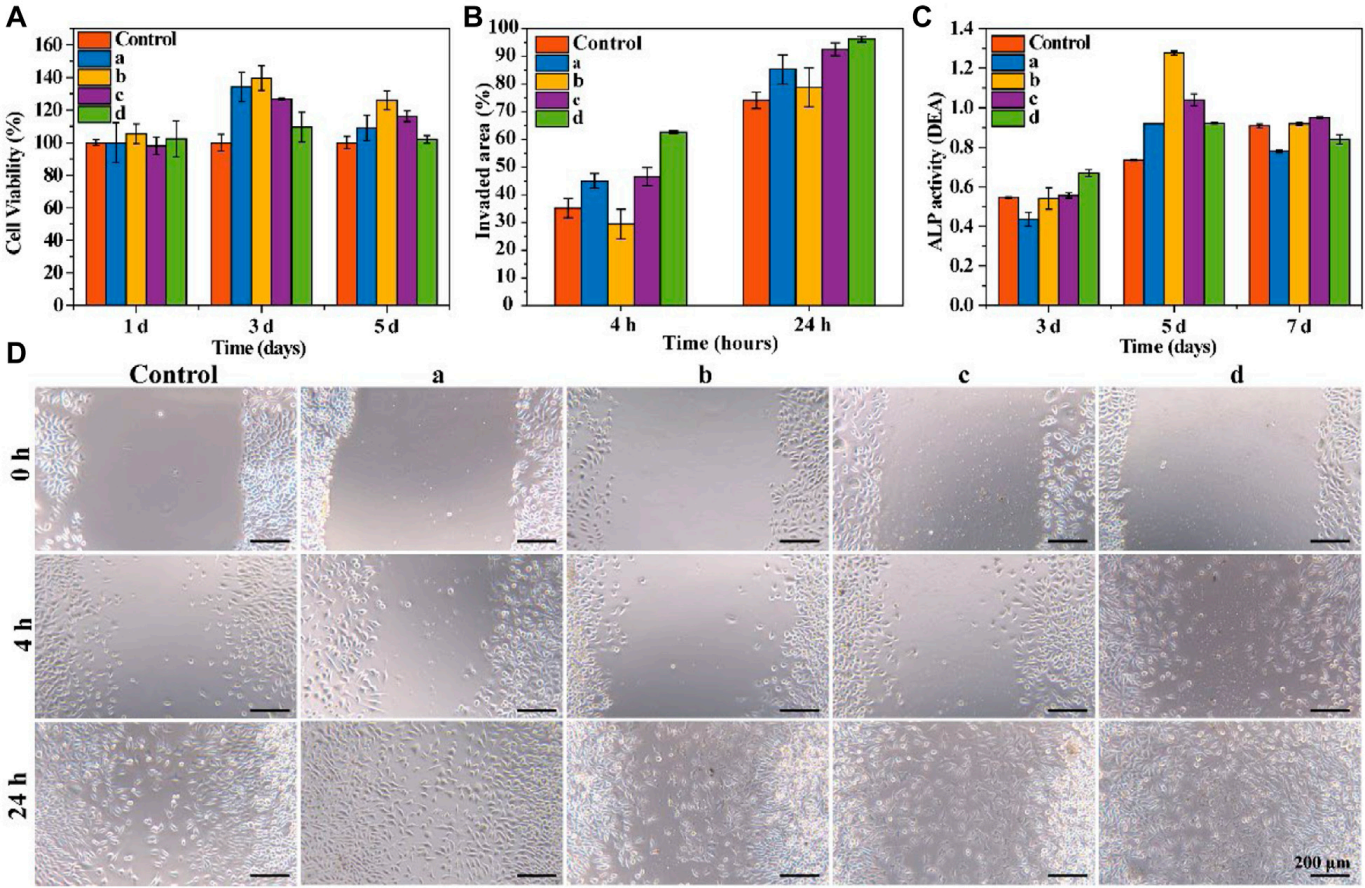
*In vitro* cytotoxicity and functional evaluation. **(A)** Cell viability of rBMSCs co-cultured with bone waxes over 7 days. **(B)** Quantitative analysis of scratch area at 4 h and 24 h **(C)** ALP activity assay of rBMSCs cultured with bone waxes at different timepoints. **(D)** Representative photographs of the migration of HUVECs treated with different bone waxes (a, b, c, and d).

The cytotoxicity of the prepared samples to rBMSCs was examined using a CCK-8 assay. All samples were completely disintegrated in the medium during the first day of culturing. As shown in Figure 3A, all bone wax groups showed significantly increased cell viability after 3 days of culture. Though bone wax d had relatively lower cell viability compared with other bone wax samples at day 3 and 5, all samples had a higher cell viability than the control group, indicating that *β*-TCP and starch microspheres were biocompatible and positively affected cell proliferation. The relatively low cell viability of bone wax d may be because the total amount of *β*-TCP and starch microspheres was higher, causing a certain toxicity to rBMSCs and affecting cell proliferation, but overall cell viability was still greater than 80% and no cytotoxicity was observed.

### 3.4 Bone wax promotion of cell migration and osteogenesis

To examine the effect of materials on cell migration, a scratch wound assay was conducted. Cell migration images are shown in Figure 3D. All groups exhibited the same trend for cells invading the scratch area. After incubation for 24 h, the bone wax groups showed a significantly increased rate of HUVEC migration compared with that in control groups. Cells exposed to sample d exhibited the fastest migration rate (96.17 ± 0.94%), followed by those exposed to sample c (92.49 ± 2.33%), sample a (85.33 ± 5.29%), sample b (78.78 ± 7.04%), and the control (74.13 ± 2.97%) (Figure 3B).

Alkaline phosphatase (ALP) activity was used to evaluate the osteogenesis effect of resorbable bone waxes (Figure 3C). As culture time increased from day 3 to day 5, ALP activity also increased. On day 3, rBMSCs co-cultured with bone wax d showed the highest ALP activity, indicating that *β*-TCP could induce osteogenic differentiation. When cultured for more than 5 days, the ALP activity of bone wax d was not the highest of the samples, perhaps due to the faster proliferation of cells cultured in bone waxes b and c.

### 3.5 Evaluation of the bone healing promotion effect *in vivo*

**FIGURE 4:**
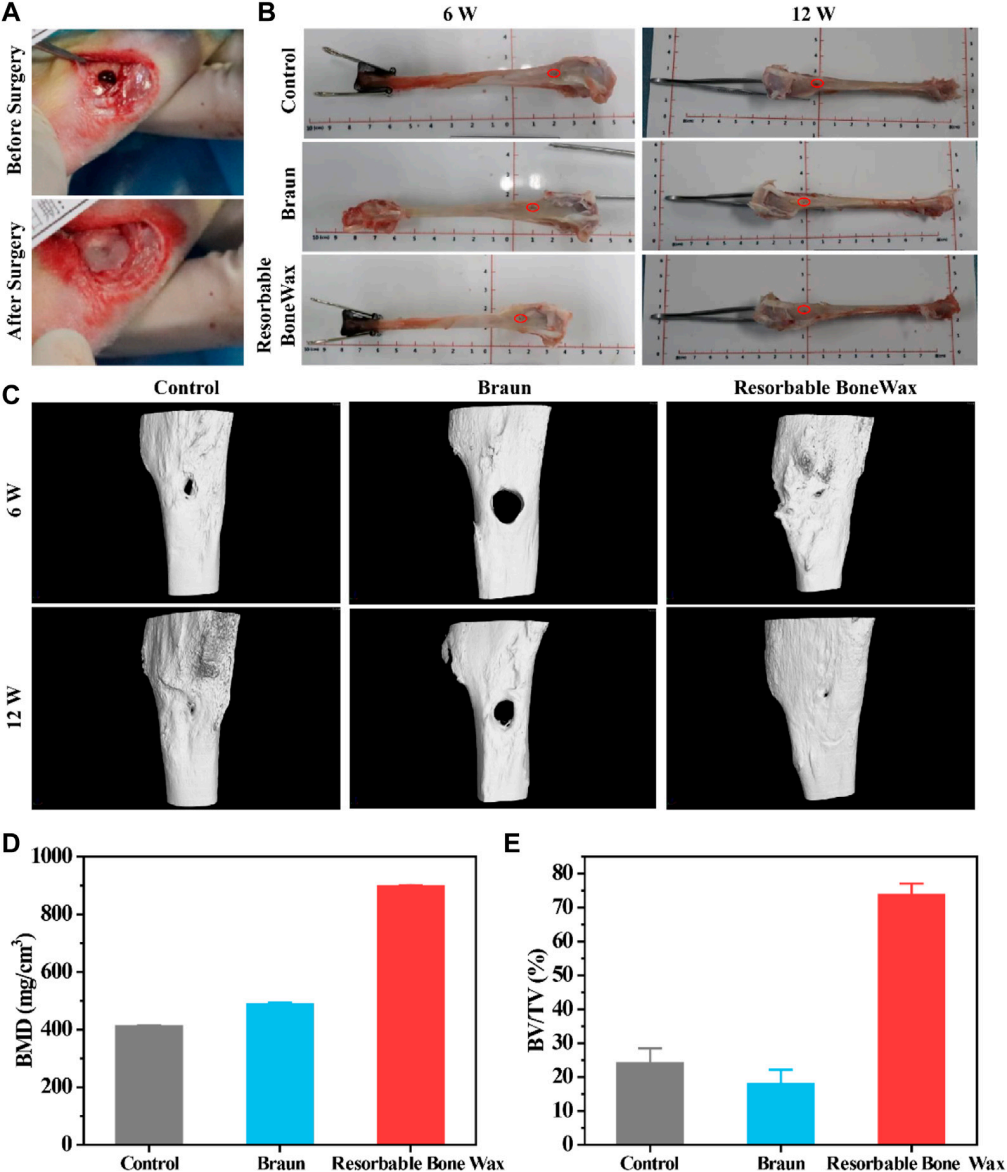
*In vivo* bone formation ability assessment **(A)** Surgical procedure for tibial injury and bone wax application. **(B)** Images of tibia 6 and 12 weeks after surgery. **(C)** Micro-CT images of tibia 6 and 12 weeks after surgery. **(D)** Statistical calculation of bone density from CT. **(E)** Statistical calculation of BV/TV from CT.

A tibia defect model was conducted in rabbit to assess the bone healing effect, and commercial B. Braun Bone Wax was used as a positive control group. Resorbable bone wax d was chosen as the experimental sample and marked as resorbable bone wax in the following experiment. The bone waxes were used to seal the injury site, and representative surgery images are shown in Figure 4A.

Hemostasis evaluation was performed by assessing local hemostasis time and bleeding volume when the bone waxes were applied to the defects. As shown in Table 2, hemostasis time of the control group treated by medical gauze was 250 ± 92 s, and the bleeding volume was 1.46 ± 1.25 g. When treated with ether Braun or resorbable bone wax, the bleeding stopped as soon as the bone wax was pressed against the defects and no hemorrhaging was observed after 5 min. The hemostatic effect of resorbable bone wax was consistent with Braun bone wax.

**TABLE 2:** Hemostasis time and bleeding volume of animals.

Groups	Hemostasis time (s)	Bleeding volume (g)
Control	250 ± 92	1.46 ± 1.25
Resorbable bone wax d	0 ± 0	0.00 ± 0.00
B. Braun bone wax	0 ± 0	0.00 ± 0.00

Subsequently, we evaluated bone formation ability, and tibia at 6 and 12 weeks were harvested for micro-CT and histological staining evaluation. Tibia images at weeks 6 and 12 are shown in Figure 4B and the defect site is marked in red.

Representative micro-CT images of defective tibia are shown in Figure 4C. Osteogenesis was observed in all groups and defects significantly decreased in size. Prominent new bone formation was also found in the control group, although the small bone defect was still present at 12 weeks. The remaining defect in the commercial Braun group had the highest volume area. Additionally, thick yellow matter, which was probably residual bone wax, was found at the defect site in the Braun group after gross examination at 6 weeks. A similar phenomenon has also been reported by research published previously, in which bone wax was observed to remain at the original site or migrate to a different location after implantation (Kim et al., 2020). The resorbable bone wax-treated group showed the fastest bone formation; defects were nearly completely repaired 12 weeks after surgery. Besides, resorbable bone wax was completely absorbed 6 weeks post-surgery. The resorbable bone wax-treated group exhibited the highest bone mineral density (BMD) (896.6 ± 4.12 mg/cm^3^), almost double that of the other two groups (Figure 4D). The bone volume fraction (BV/TV) of the resorbable bone wax-treated group reached 73.79 ± 3.24%. The Braun bone wax hindered bone formation and had a BV/TV (18.02 ± 4.12%) that was lower than the control group (24.12 ± 4.32%) (Figure 4E). The results indicate the adverse effect on bone healing of non-resorbable bone wax and the beneficial effect on bone regeneration of resorbable bone wax.

**FIGURE 5:**
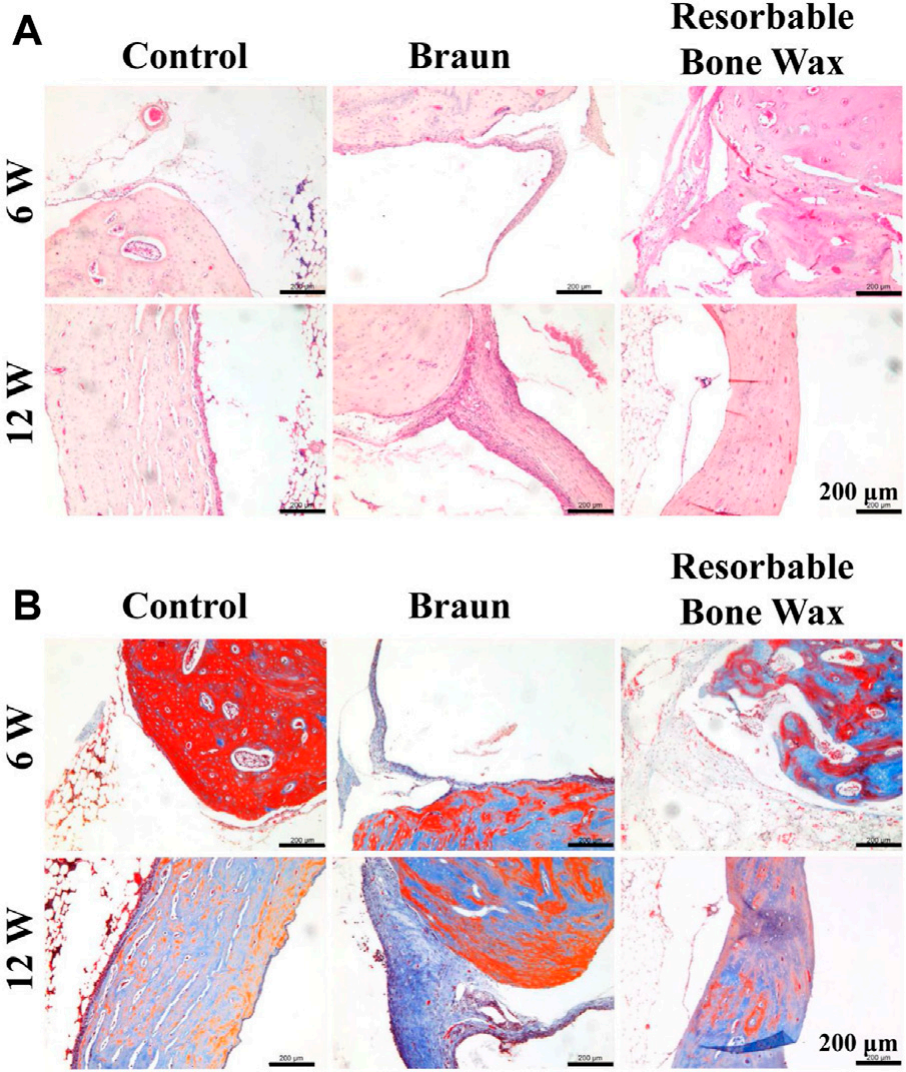
Histological staining of tibia. **(A, B)** H&E **(A)** and Masson **(B)** staining images at defect sites 6 and 12 weeks post-surgery.

Histological analysis was performed to further analyze bone regeneration. H&E staining images confirmed the micro-CT observations (Figure 5A). In the control and resorbable bone wax groups no residual substance was found at the bone defect surface, and new bone formation was observed in the defect area 6 weeks after implantation. Apparent bone formation and defect repair was observed at 12 weeks. The regeneration of bone defects in the resorbable bone wax group was significantly better than in the Braun bone wax group. By contrast, bone wax particles and fibrotic tissue appeared in the Braun group at both 6 and 12 weeks. Only a small amount of new bone formation was observed in the Braun group.

Masson staining was performed to identify the collagen formation of new bone (Figure 5B). At 6 weeks post-surgery, blue-stained type 1 collagen was obvious in the control and resorbable bone wax groups, suggesting the early stage of bone repair. In the Braun group, unfilled bone defect was surrounded by fibrous tissue. After 12 weeks, extensive mature bone structure (stained red) confirmed bone healing in the control and resorbable bone wax groups, whereas fibrous tissue and a small amount of newly formed bone occupied the defect area in the Braun group.

**FIGURE 6:**
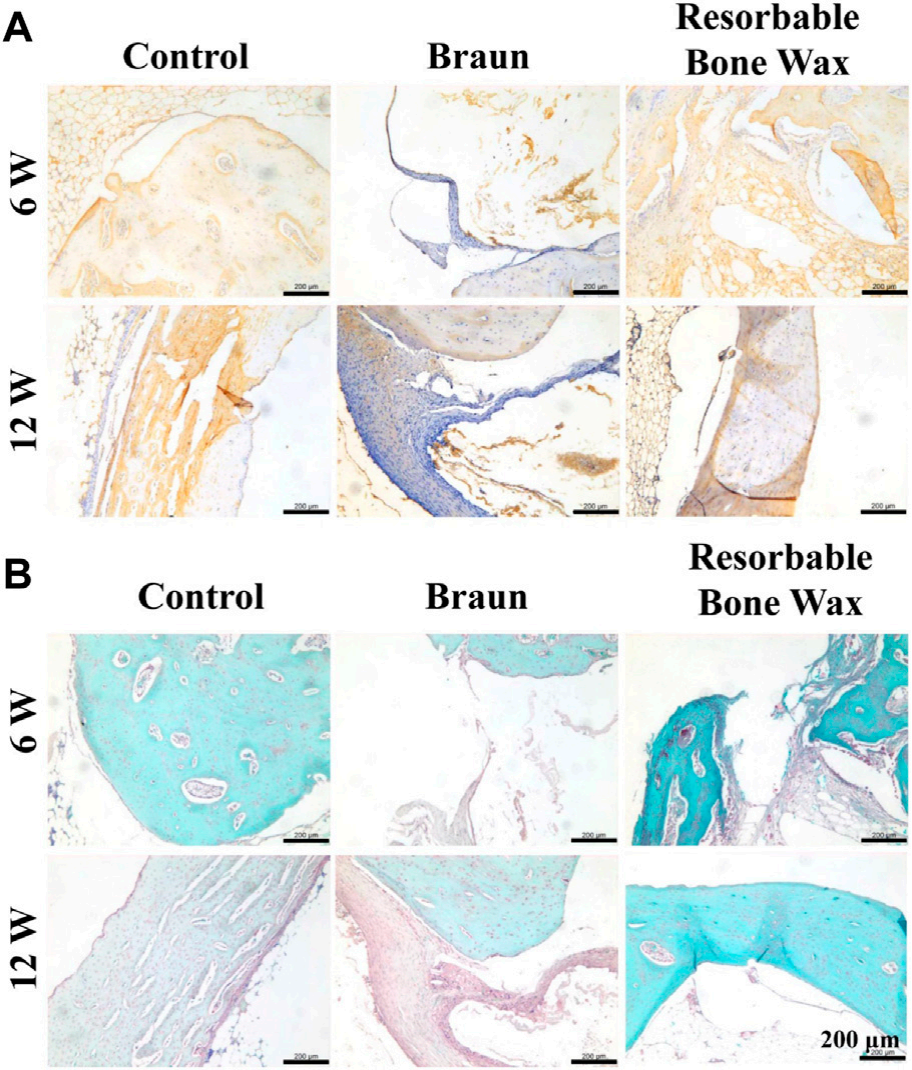
Histological staining of tibia. **(A, B)** Immunohistochemistry staining of OCN **(A)** and safranin O and Fast Green staining images **(B)** at defect sites 6 and 12 weeks post-surgery.

Osteocalcin (OCN), which is a protein synthesized by the osteoblast, has been used as a marker of bone mineralization since its discovery (Rubert and Piedra, 2020). Immunohistochemistry staining of OCN is shown in Figure 6A. OCN was highly expressed in the control and resorbable bone wax groups 6 weeks postoperatively and then was decreased at 12 weeks. In the Braun bone wax group, the expression of OCN was suppressed until 12 weeks after surgery, which might be due to the limited amount of new bone formation.

Safranin O and Fast Green staining was carried out after 6 and 12 weeks to evaluate bone formation. As shown in Figure 6B, new bone (green) appeared in all groups at 6 weeks. At 12 weeks post-surgery, massive new bone formation was found in the control and resorbable bone wax groups, whereas the defect in the Braun group was still in the process of reparation, with only a small amount of new bone formation observed.

## 4 Discussion

Owing to the complex vascular structure in bones, severe osseous haemorrhage is difficult to control with natural hemostasis. To avoid further tissue necrosis and eventual mortality (Hickman et al., 2017), bone wax has been widely introduced in clinical environments as an essential hemostatic agent that works by mechanically sealing the bleeding site (Zhou et al., 2019b). Bone wax is well known for its cost-effectiveness and ease of operation (Moo et al., 2016). However, conventional bone wax, which mostly comprises beeswax softened with paraffin or isopropyl palmitate, or both (Das, 2018), is non-resorbable and causes numerous complications (Chun et al., 1988; Eser et al., 2007; Lavigne et al., 2008; Katre et al., 2010; Ozerdem et al., 2013; Choi and Yang, 2017; Baird et al., 2018; Fahradyan et al., 2018; Zhou et al., 2019a; Thangamathesvaran et al., 2019). Reports of the development of novel resorbable bone wax substitutes became common during these years. Alkylene oxide copolymer-based hemostatic bone wax substitute was first reported in 2001 by Wang et al. (Wang et al., 2001). The water-soluble material proved to be effective in hemostasis, could be easily handled, and was fully resorbable within 24–48 h. Suwanprateeb et al. (Suwanprateeb et al., 2014) increased the liquid sealing duration and workability of a similar alkylene oxide copolymer material by adding pregelatinized starch.

In this study, we aimed to prepare a novel bone wax formulation by mixing alkylene oxide copolymers with *β*-TCP and starch microspheres to promote hemostasis and bone regeneration. *β*-TCP and starch microspheres were successfully synthesized and four different bone waxes were prepared for further selection (Figure 1). *In vitro* and *in vivo* evaluations confirmed that starch microspheres and *β*-TCP were biodegradable, biocompatible, and hemostatically effective (Figures 2, 3), consistent with a previous report (Li et al., 2019). Furthermore, *β*-TCP can form negative potential on the microsphere surface, which accelerates blood clotting time (Li et al., 2016; Naudin et al., 2017). The addition of these two materials improved the biocompatibility (Figure 3A) and hemostatic performance of resorbable bone wax (Table 2). In terms of cost performance, we assessed the benefit of the composite of *β*-TCP or starch microspheres, or both, with caution.

As expected, the results of *in vitro* experiments proved that the addition of *β*-TCP and starch microspheres significantly improve the biocompatibility and bioactivity of bone wax without any negative effect on its physical properties (Figures 2, 3). Resorbable bone wax d was the most promising formulation of resorbable bone wax and warranted further *in vivo* assessment in this study. The *in vivo* assessment showed that resorbable bone wax has the same effective hemostatic performance as B. Braun Bone Wax. Additionally, the resorbable bone wax can be fully resorbed in 6 weeks, avoiding residues that hinder bone regeneration *in vivo*. Micro CT images and further histological staining of bone defect sites indicated that the resorbable bone wax does not affect the rate of bone regeneration after sealing (Figure 4). Owing to the addition of *β*-TCP, the resorbable bone wax-treated group showed higher OCN expression (Figure 6A). The bone tissue can basically recover completely 12 weeks after surgery, and resorbable bone wax, unlike traditional non-resorbable bone wax, will not cause non-union of the bone tissue.

## 5 Conclusion

Novel resorbable bone waxes based on alkylene oxide copolymers, *β*-TCP, and starch microspheres, were prepared for bone hemostasis and healing. Bone bleeding can be immediately stopped after the application of bone wax, which showed the same hemostatic effect as B. Braun Bone Wax. Additionally, resorbable bone wax, which can be thoroughly resorbed in 6 weeks, exhibits faster absorption compared with Braun bone wax. The results of *in vitro* and *in vivo* assessments demonstrated the bioactivity, biocompatibility, and effective hemostatic performance of the novel resorbable bone wax. The regeneration of a rabbit tibia defect applied with resorbable bone wax was significantly improved compared with the same defect treated with non-resorbable bone wax. Thus, resorbable bone wax has the potential to be a substitute for conventional bone wax in bone hemostasis.

## Data Availability

The original contributions presented in the study are included in the article/Supplementary Material, further inquiries can be directed to the corresponding authors.

## References

[B1] Achneck H. E. Sileshi B. Jamiolkowski R. M. Albala D. M. Shapiro M. L. Lawson J. H. (2010). A comprehensive review of topical hemostatic agents: Efficacy and recommendations for use. Ann. Surg. 251 (2), 217–228. 10.1097/sla.0b013e3181c3bcca 20010084

[B2] Baird S. M. Teh B. M. Lim K. K. Campbell M. C. (2018). Bone wax extrusion through postauricular wounds: A case series. Laryngoscope 128 (2), 369–372. 10.1002/lary.26697 28561384

[B3] Björses K. Faxälv L. Montan C. Wildt-Persson K. Fyhr P. Holst J. (2011). *In vitro* and *in vivo* evaluation of chemically modified degradable starch microspheres for topical haemostasis. Acta Biomater. 7 (6), 2558–2565. 10.1016/j.actbio.2011.03.003 21382526

[B4] Buser D. Hoffmann B. Bernard J. i. Lussi A. Schenk R. K. (2010). Evaluation of filling materials in membrane--protected bone defects. A comparative histomorphometric study in the mandible of miniature pigs. Clin. Oral Implants Res. 9 (3), 137–150. 10.1034/j.1600-0501.1998.090301.x 10530128

[B5] Chao D. Xu M. Yin Y. Pu J. (2017). Preparation and characterization of hydrophobic non-crystal microporous starch (NCMS) and its application in food wrapper paper as a sizing agent. Bioresources 12 (3), 5775–5789.

[B6] Chen C. Li H. Pan J. Yan Z. Yao Z. Fan W. (2015). Biodegradable composite scaffolds of bioactive glass/chitosan/carboxymethyl cellulose for hemostatic and bone regeneration. Biotechnol. Lett. 37 (2), 457–465. 10.1007/s10529-014-1697-9 25326173

[B7] Choi B. K. Yang E. J. (2017). Delayed infection after using bone wax in maxillofacial surgery: A rare complication after reduction mandibuloplasty. Wound Med. 17, 18–23. 10.1016/j.wndm.2017.01.004

[B8] Choi S. Y. Rhim J. Heo S. A. Han W. J. Kim M. H. Ha C. W. (2021). Efficacy and safety of a novel hemostatic material, BoneStat, compared with Ostene and Bone Wax in a rat calvarial defect model. Int. J. Artif. Organs 44 (10), 734–747. 10.1177/03913988211021428 34387533

[B9] Chun P. Virmani R. Mason T. E. Johnson F. (1988). Bone wax granuloma causing saphenous vein graft thrombosis. Am. Heart J. 115 (6), 1310–1313. 10.1016/0002-8703(88)90029-4 3287874

[B10] Das J. M. (2018). Bone wax in neurosurgery: A review. World Neurosurg. 116, 72–76. 10.1016/j.wneu.2018.04.222 29753076

[B11] Eser O. Cosar M. Aslan A. Sahin O. (2007). Bone wax as a cause of foreign body reaction after lumbar disc surgery: A case report. Adv. Ther. 24 (3), 594–597. 10.1007/bf02848783 17660169

[B12] Fahradyan A. Ohanisian L. Tsuha M. Park M. J. Hammoudeh J. A. (2018). An unusual complication of bone wax utilization. J. Craniofacial Surg. 29 (4), 976–979. 10.1097/scs.0000000000004321 29438209

[B13] Grigoraviciute-Puroniene I. Tsuru K. Garskaite E. Stankeviciute Z. Beganskiene A. Ishikawa K. (2017). A novel wet polymeric precipitation synthesis method for monophasic β-TCP. Adv. Powder Technol. 28 (9), 2325–2331. 10.1016/j.apt.2017.06.014

[B14] Hickman D. Pawlowski C. L. Sekhon U. Marks J. Gupta A. S. (2017). Biomaterials and advanced technologies for hemostatic management of bleeding. Adv. Mater. 30 (4), 1804635. 10.1002/adma.201804635 PMC5831165 29164804

[B15] Katre C. Triantafyllou A. Shaw R. J. Brown J. S. (2010). Inferior alveolar nerve damage caused by bone wax in third molar surgery. Int. J. Oral Maxillofac. Surg. 39 (5), 511–513. 10.1016/j.ijom.2009.06.031 20382504

[B16] Kim H. E. Yoon H. Y. Kim E. J. Kim S. J. (2020). Effects of poly (ethylene glycol-propylene glycol) copolymer on hemostasis and osteogenesis in a rat calvarial defect model. Korean J. Veterinary Res. 60 (3), 145–153. 10.14405/kjvr.2020.60.3.145

[B17] Lavigne M. Rama K. Doyon J. Vendittoli P. A. (2008). Bone-wax granuloma after femoral neck osteoplasty, Canadian journal of surgery. J. Can. de Chir. 51 (3), E58–E60. PMC2496602 18682762

[B18] Li B. Z. Wang L. J. Li D. Chiu Y. L. Zhang Z. J. Shi J. (2009). Physical properties and loading capacity of starch-based microparticles crosslinked with trisodium trimetaphosphate. J. Food Eng. 92 (3), 255–260. 10.1016/j.jfoodeng.2008.10.008

[B19] Li G. Quan K. Liang Y. Li T. Yuan Q. Tao L. (2016). Graphene-montmorillonite composite sponge for safe and effective hemostasis. Acs Appl. Mater. Interfaces 8 (51), 35071–35080. 10.1021/acsami.6b13302 27935296

[B20] Li Q. Lu F. Shang S. Ye H. Yu K. Lu B. (2019). Biodegradable microporous starch with assembled thrombin for rapid induction of hemostasis. ACS Sustain. Chem. Eng. 7 (10), 9121–9132. 10.1021/acssuschemeng.8b05701

[B21] Malafaya P. B. Silva G. A. Reis R. L. (2007). Natural-origin polymers as carriers and scaffolds for biomolecules and cell delivery in tissue engineering applications. Adv. drug Deliv. Rev. 59 (4-5), 207–233. 10.1016/j.addr.2007.03.012 17482309

[B22] Michael J. Grindel T. Jaworski R. Martin E. Paula C. (2002). Pharmacokinetics of a novel surface-active agent, purified poloxamer 188, in rat, rabbit, dog and man. Biopharm. Drug Dispos. 23 (3), 87–103. 10.1002/bdd.297 12173548

[B23] Moo I. H. Chen J. Pagkaliwaga E. H. Tan S. W. Poon K. B. (2016). Bone wax is effective in reducing blood loss after total knee arthroplasty. J. Arthroplasty 32 (5), 1483–1487. 10.1016/j.arth.2016.12.028 28089184

[B24] Naudin C. Burillo E. Blankenberg S. Butler L. Renné T. (2017). Factor XII contact activation. Seminars Thrombosis Hemostasis 43 (08), 814–826. 10.1055/s-0036-1598003 28346966

[B25] Ogose A. Hotta T. Kawashima H. Kondo N. Gu W. Kamura T. (2005). Comparison of hydroxyapatite and beta tricalcium phosphate as bone substitutes after excision of bone tumors. J. Biomed. Mater. Res. Part B Appl. Biomaterials 72 (1), 94–101. 10.1002/jbm.b.30136 15376187

[B26] Orgill D. P. Ehret F. W. Regan J. F. Glowacki J. Mulliken J. B. (2015). Polyethylene glycol/microfibrillar collagen composite as a new resorbable hemostatic bone wax. J. Biomed. Mater. Res. 39 (3), 358–363. 10.1002/(sici)1097-4636(19980305)39:3<358::aid-jbm3>3.0.co;2-i 9468043

[B27] Ozerdem G. Hidiroglu M. Kucuker A. Kunt A. Cetin L. (2013). Bone wax as a cause of a foreign body granuloma in a resternotomy: A case report. J. Cardiothorac. Surg. 8 (1), P121. 10.1186/1749-8090-8-s1-p121

[B28] Peng H. Xiong H. Wang S. Li J. Chen L. Zhao Q. (2011). Soluble starch–based biodegradable and microporous microspheres as potential adsorbent for stabilization and controlled release of coix seed oil. Eur. Food Res. Technol. 232 (4), 693–702. 10.1007/s00217-011-1438-4

[B29] Rubert M. Piedra C. (2020). La osteocalcina: De marcador de formación ósea a hormona; y el hueso, un órgano endocrino. Rev. Osteoporosis Metab. Miner. 12 (4), 146–151. 10.4321/s1889-836x2020000400007

[B30] Salim H. A. (2018). Comparison of Ostene® and bone wax on bone healing: A comparative experimental study in rabbits. J. Oral Res. 7 (9), 362–367. 10.17126/joralres.2018.082

[B31] Schonauer C. Tessitore E. Barbagallo G. Albanese V. Moraci A. (2004). The use of local agents: Bone wax, gelatin, collagen, oxidized cellulose. Eur. Spine J. 13 (1), S89–S96. 10.1007/s00586-004-0727-z 15221572 PMC3592193

[B32] Suwanprateeb J. Kiertkrittikhoon S. Kintarak J. Suvannapruk W. Thammarakcharoen F. Rukskul P. (2014). *In vivo* assessment of new resorbable PEG–PPG–PEG copolymer/starch bone wax in bone healing and tissue reaction of bone defect in rabbit model. J. Mater. Sci. Mater. Med. 25 (9), 2131–2139. 10.1007/s10856-014-5249-6 24913421

[B33] Suwanprateeb J. Suvannapruk W. Thammarakcharoen F. Chokevivat W. Rukskul P. (2013). Preparation and characterization of PEG–PPG–PEG copolymer/pregelatinized starch blends for use as resorbable bone hemostatic wax. J. Mater. Sci. Mater. Med. 24 (12), 2881–2888. 10.1007/s10856-013-5027-x 23955721

[B34] Tangboriboon N. Suttiprapar J. Changkhamchom S. Sirivat A. (2019). Alternative green preparation of mesoporous calcium hydroxyapatite by chemical reaction of eggshell and phosphoric acid. Int. J. Appl. Ceram. Technol. 16 (5), 1989–1997. 10.1111/ijac.13241

[B35] Tham T. Roberts K. Shanahan J. Burban J. Costantino P. (2018). Analysis of bone healing with a novel bone wax substitute compared with bone wax in a porcine bone defect model. Future Sci. OA 4 (8), FSO326. 10.4155/fsoa-2018-0004 30271614 PMC6153452

[B36] Thangamathesvaran L. Mirani N. Turbin R. Langer P. D. (2019). Chronic, symptomatic orbital inflammation resulting from retained bone wax. Ophthalmic Plastic Reconstr. Surg. 35 (6), e147–e148. 10.1097/iop.0000000000001471 31593040

[B37] Wang M. Y. Armstrong J. K. Fisher T. C. Meiselman H. J. Mccomb G. J. Levy M. L. (2001). A new, pluronic-based, bone hemostatic agent that does not impair osteogenesis. Neurosurgery 49 (4), 962–968. 10.1097/00006123-200110000-00031 11564259

[B38] Wang Z. Telci D. Griffin M. (2011). Importance of syndecan-4 and syndecan -2 in osteoblast cell adhesion and survival mediated by a tissue transglutaminase-fibronectin complex. Exp. Cell. Res. 317 (3), 367–381. 10.1016/j.yexcr.2010.10.015 21036168

[B39] Yu H. Yan X. Pan Y. Tang S. Sun X. Yang X. (2017). Influences of mesoporous zinc-calcium silicate on water absorption, degradability, antibacterial efficacy, hemostatic performances and cell viability to microporous starch based hemostat. Mater. Sci. Eng. C 76, 340–349. 10.1016/j.msec.2017.03.094 28482536

[B40] Zhou H. Ge J. Bai Y. Liang C. Yang L. (2019). Translation of bone wax and its substitutes: History, clinical status and future directions. J. Orthop. Transl. 17, 64–72. 10.1016/j.jot.2019.03.005 PMC6551357 31194062

[B41] Zhou H. Yang M. Ni X. Yang L. Kutty M. G. (2018). Using calcium sulfate cement—hydroxypropyl methyl cellulose/sodium alginate composites as substitutes of bone wax. Int. J. Appl. Ceram. Technol. 15 (4), 903–909. 10.1111/ijac.12839

[B42] Zhou Y. Li M. Wei X. Yang X. Zhang J. Qi X. (2019). Bone wax migrates to the orbit in a patient with a frontal sinus abnormality: A case report. BMC Ophthalmol. 19 (1), 29–36. 10.1186/s12886-019-1037-x 30678648 PMC6345031

